# Epithelial Cancer

**Published:** 1855-09

**Authors:** William Van Pelt

**Affiliations:** Williamsville, Erie Co., N. Y.


### ART. V.—*Epithelial Cancer.* By WILLIAM VAN PELT, Williamsville, Erie Co., N. Y.

One of the oldest residents in our town, Mr. James Young, consulted me last autumn in regard to an epithelial cancer, upon the dorsum of the left hand. I advised amputation; but that he declined, making the request that I would make the cure in some other manner. Accordingly escharotics were applied, without producing any improvement; and becoming satisfied that nothing could be gained by a farther application of them, I desisted. Some fourteen years since I remembered to have seen a wart upon his hand and then advised excision. It then had the appearance of an ordinary wart, and at that time it gave no inconvenience other than its liability to injury, incident to its location. Frequent irritation gradually promoted its development. On the 30th of March, 1855, he requested me to perform amputation, having submitted to treatment by certain noted irregular practitioners, who make cancroid affections a specialty. The hand now presented an irregular morbid growth, with an ulcerated surface, measuring more than two and three-fourths inches in diameter; edges elevated and involuted; granulations pale and coarse, interspersed with cells in every stage of development; the discharge was profuse. The patient complained of a burning pain, and was considerably emaciated. Three days after, in the presence of and with the assistance of my friend, Dr. Ham, I performed the amputation. The patient desired the administration of chloroform, which I refused, thinking his age might render apoplexy more liable, than its omission. Having in consideration the opinion of M. Lebert, and others, that the parts in proximity to the diseased integument, were equally infiltrated with the epidermic cells, and being therefore rendered prone to a relapse, therefore the point on the forearm, three inches below the elbow, was chosen for separation. I resorted to the flap operation, making the greater one before. But two vessels required ligation. The edges of the flaps were retained by sutures and adhesive straps, and the usual dressings.

On the twenty-second day after, the last ligature came away; and no untoward circumstance retarded the healing of the stump. At this time, one hundred and twelve days, he appears in sound health, with no return of the affection, and although seventy-five years old, looks much younger.

I think this instance will furnish additional evidence that Lawrence's opinion is true in regard to this form of disease, viz: "that it is locally malignant; but constitutionally innocent.'' Though so mild in the beginning, it acquires in time an energy which, by slow integration, destroys the firmest tissues of the body, as in this case the metacarpal bones of the first and second finger were severed. If epithelial cancer may be regarded as depraved action in the dermal structure, and seldom followed by secondary deposits, like the primary morbid growth; the surgeon may very properly give assurance of a medical cure, by amputation, when favorably.

